# A Case-Control Study to Identify Risk Factors Associated with Avian Influenza Subtype H9N2 on Commercial Poultry Farms in Pakistan

**DOI:** 10.1371/journal.pone.0119019

**Published:** 2015-03-16

**Authors:** Mamoona Chaudhry, Hamad B. Rashid, Michael Thrusfield, Sue Welburn, Barend MdeC. Bronsvoort

**Affiliations:** 1 Division of Infection and Pathway Medicine, The University of Edinburgh Medical School, Edinburgh, Scotland, United Kingdom; 2 Department of Clinical Medicine and Surgery, University of Veterinary and Animal Sciences, Abdul Qadir Jilani Road, Lahore, Pakistan; 3 The Royal (Dick) School of Veterinary Studies, The University of Edinburgh, Easter Bush, Roslin, Midlothian, Edinburgh, Scotland, United Kingdom; 4 The University of Edinburgh, Roslin Institute at the R(D)SVS, Easter Bush, Roslin, Midlothian, Edinburgh, Scotland, United Kingdom; University of Maryland, UNITED STATES

## Abstract

A 1:1 matched case-control study was conducted to identify risk factors for avian influenza subtype H9N2 infection on commercial poultry farms in 16 districts of Punjab, and 1 administrative unit of Pakistan. One hundred and thirty-three laboratory confirmed positive case farms were matched on the date of sample submission with 133 negative control farms. The association between a series of farm-level characteristics and the presence or absence of H9N2 was assessed by univariable analysis. Characteristics associated with H9N2 risk that passed the initial screening were included in a multivariable conditional logistic regression model. Manual and automated approaches were used, which produced similar models. Key risk factors from all approaches included selling of eggs/birds directly to live bird retail stalls, being near case/infected farms, a previous history of infectious bursal disease (IBD) on the farm and having cover on the water storage tanks. The findings of current study are in line with results of many other studies conducted in various countries to identify similar risk factors for AI subtype H9N2 infection. Enhancing protective measures and controlling risks identified in this study could reduce spread of AI subtype H9N2 and other AI viruses between poultry farms in Pakistan.

## Introduction

Avian influenza virus (AIV) A subtype H9N2 has become prevalent in domestic poultry in many countries in Asia and The Middle East since the late 1990’s [1]. Outbreaks of AIV subtype H9N2 in commercial chickens have been reported in Iran (1998), Pakistan (1998), China (1994), Korea (1996), United Arab Emirates (2000–2003), Israel (2000–2006), Jordan (2003), Lebanon (2004), and Iraq (2004–2007) [2, 3].

According to FAO [4] there are four sectors of poultry production systems (i.e Industrial integrated system with high levels of biosecurity; commercial poultry production system with moderate to high biosecurity; commercial poultry production system with low to minimal biosecurity; and village or backyard production system with minimal biosecurity). Pakistan has all four sectors of poultry production system as mentioned above. In Pakistan, poultry production contributes 35% of livestock production and it has become the second largest industry after cotton, with an annual turnover of US$ 2 billion. The poultry sector has proved to be one of the most vibrant segments of the agriculture sector in Pakistan. Directly and indirectly, 1.5 million people are estimated to have benefitted from this sector in terms of employment and income [5].

Avian influenza outbreaks have a devastating impact on these commercial poultry sectors in Pakistan and many outbreaks of AIV e.g. subtype H7N3 (three outbreaks in 1995, 1998, 2001–2002), H5N1 (three outbreaks, 2006–2008), and H9N2 (first time reported in 1998, since then it has become endemic in the country) have been reported in Pakistan [6–9]. Despite the endemicity and losses in the poultry sector from avian influenza, particularly subtype H9N2, there is little information on risk factors for outbreaks or the use of biosecurity measures by poultry farmers in Pakistan.

Identification and quantification of locally important risk factors associated with infected farms is a critically important step in the development of risk-based surveillance and control strategies. Many published articles have quantified different risk factors for various subtypes of avian influenza in commercial poultry farms all over the world [10–23]. Previously published studies have demonstrated that avian influenza introduction, transmission and persistence are associated with poultry trading pattern [12, 20], human and poultry densities [18, 23], movement of human and fomites [10, 12, 14], low biosecurity [10, 13, 21, 22], proximity to water bodies [15, 16, 17, 23], distance from other commercial poultry farms [11, 13, 21], and proximity to roads [15, 16, 19]. These risk factors help to further identify high-risk farms/systems, which could be targeted for interventions such as vaccination or culling. Removal of identified risk factors plays an important role in the control of disease burden. However, no work has been done to quantify risk factors associated with infection of AIV in commercial poultry sector of Pakistan using analytical epidemiological techniques. Knowledge of the epidemiology of AIV (especially H9N2) in Pakistan is also inadequate. Bearing in mind the importance of poultry production system in Pakistan, which provides a good source of protein, and importance of the epidemiology of avian influenza in birds, a major threat for veterinary and public health, the following study was designed to identify and quantify risk factors associated with the presence of AIV subtype H9N2 on commercial poultry farms of Pakistan. These findings may also provide insight into the mechanism of spread of AIV subtype H9N2 in Asia.

## Materials and Methods

### Study Design

The eligible population was commercial poultry farms producing poultry/products for commercial market and consumption in Pakistan and source population of this study consisted of all types of poultry raising premises in 16 districts of the Punjab and 1 administrative unit i.e. Capital city of Pakistan. The final study population was commercial poultry farms submitting samples for laboratory analysis to the collaborating commercial laboratory from these areas. There were approximately 19,713 broiler farms and 3,599 layer farms in the study area [24]. A matched, 1:1, case-control design was used. A case farm was defined as a commercial poultry farm, which had submitted samples (lung, intestine, trachea) to the laboratory for diagnosis of H9N2 between May 2009 and January 2010 and which were confirmed as positive through virus isolation and sub-typed as H9N2 by hemagglutination inhibition test (HI). Routine monitoring of flocks at commercial farm is conducted by the flock managers. Daily record of disease data i.e. morbidity and mortality is maintained at farm level. When a farm manager suspects any sign of influenza like illness in the flock, samples are immediately dispatched to the commercial or public laboratories for post mortem examination or further laboratory investigations. The laboratory investigations in the current study were carried out by a private laboratory providing diagnostic services to commercial poultry farmers. The samples were initially screened by postmortem examination of dead birds. Positive samples were further processed by virus isolation and subtyping by hemagglutination inhibition test. Laboratory provided the data on request of the research team. A control farm was defined as any commercial farm, which submitted samples (feces/blood) to the same laboratory for diagnosis of parasitic diseases (the other major diagnostic category requested other than for AI) and were matched to cases based on month of submission. Control farms needed to have the potential to be cases so only farms submitting samples were considered for controls on the assumption that given they were prepared to submit samples they would be more likely to participate in the study. Our other intention here was to select for controls a group of farms where the mangers of the flock on those farms had a relatively good understanding of disease. Due to availability of limited funds to confirm the negative status of each control (which would have required large within flock samples) and instead the clinical records of the flock were relied upon. Based on the flock history, lack of clinical signs of influenza or influenza like infections and an average flock mortality of less than 3%, commercial farm was considered as not likely to have current AIV infection and retained as a control farm.

To achieve 80% power to detect an odds ratio of >2.0 with 95% confidence interval, assuming 40% exposure in the controls, 133 case farms and 133 control farms were required based on WINPEPI 8.7 [25, 26].

There was no requirement for ethical review to collect questionnaire-based data about management of commercial poultry flock in Pakistan. Permission was sought from the local Veterinary Officers (VOs) to contact selected owners. Owners were then contacted initially by mobile phone and the project objectives explained and they were then asked to participate in the study. If they agreed the farm was visited, a structured interview using a standardized questionnaire tool was conducted to gather information on risk factors.

The address of each farm was obtained from the logbook of the laboratory. The 266 selected farms were visited between May 2009 and January 2010 and structured questionnaire completed with the owner/farm manager at a face-to-face interview with the field team (MC and HBR). The questionnaire included 37 risk factors about farm management, biosecurity measures, location of farm, and flock history (S1 Appendix). The risk factors were selected after extensive review of published articles and knowledge of the local farming practices [10–23, 27–38]. The study was carried out for approximately 8 months. More then 80% of the case farms were visited within a month of the date of diagnosis, as they were located in the vicinity districts of the capital of Punjab. Approximately 20% farms were visited over a month from the date of diagnosis due to their remote location.

The location of each farm was recorded with a hand-held Global Positioning system (GPS, Garmin, Olathe, KS, USA) in WGS-84 datum. Maps were generated in ArcGIS 10 (Geographical Information System, ESRI System, Redlands, CA, USA). Geographical data of Pakistan boundaries, administrative division and other shape files were downloaded from the internet (http://www.diva-gis.org/datadown and http://www.mapcruzin.com/free-pakistan-arcgis-maps-shapefiles.htm).

### Statistical Analysis

Questionnaires were entered into EpiData software 3.1 (www.epidata.dk/download.php). Data were validated by crosschecking all the computerized records with the original hard copy of complete data. Statistical analysis was conducted using the *R* statistical software [39].

Several continuous variables relating to distances from roads or other farms were changed to binary variables to avoid problems of linearity. Boundaries for the categorization were chosen on the basis of predefined categories for the variables [40]. The predefined categories were based on knowledge about those variables from review of available literature and any threshold values given in the literature was chosen to define the categories e.g. distance from the main road (in km) was divided into two categories i.e. ≤0.5 km and >0.5 km, distance from the nearest commercial farm and distance from the nearest case/infected farm were categorized into ≤1 km and > 1 km [21, 22, 41–43]. Age at the time of submission of sample to the laboratory was also categorized into ≤50 days or >50 days because difference in age is related to immune response of the birds and its susceptibility to avian influenza [44, 45]. Categorical variables with more than 2 levels were included using dummy variables. A dummy variable adjustment method was adopted to deal with missing data on predictor variables in regression analysis [46, 47]. For each predictor with missing data, a dummy/indicator variable was created to indicate whether or not data are missing on that predictor. All such dummy/indicator variables were included as predictor in regression. Those dummy/indicator variables were coded with a constant value for missing data. For the construction of dummy/indicator variable “Nested IF function in Excel” was used. It was coded as “1” if the value was missing and “2” if the answer was “No” and “3” if the answer was “Yes”.

All biologically plausible and relevant variables from the questionnaire were screened using the *clogit* function of the *survival* package (version 2.37–7), which effectively performs a Mantel-Haenszel matched-pair analysis [48]. Using the standard screening approach suggested by Hosmer and Lemeshow [49] variables with a Wald statistic p-value of < 0.25 were passed on from the univariable analysis for use in development of the multivariable model. This subset of variables was then checked for colinearity using Spearman rank correlation using the *cor* function. Strongly correlated groups of variables that were also biologically related were collapsed into a new single variable for use in the final model selection process using principle component analysis (function *princomp*) and kmeans (function *kmeans*) clustering to identify two clusters based on these.

Multivariable models were developed using a backward manual stepwise elimination process [50] removing the one with the largest p-value respectively. If a variable was no longer statistically significant after adjustment for other variables it was removed (p-value = 0.05). Variables were retained or removed from the model after considering the Wald Statistic (or log likelihood ratio test for categorical variables with 3 or more levels) with a p-value of 0.05. The presence of confounding in the data was assessed by monitoring the estimated coefficient values and checking that they did not change by more than 10% when statistically non-significant variables were dropped from the model [49, 50]. The models derived manually were compared to those from the automated model selection procedure in the function *stepAIC* in the *MASS* package in R, which use Akaike’s information criterion [51] to trade of goodness-of-fit against model complexity.

## Results

**Fig 1:**
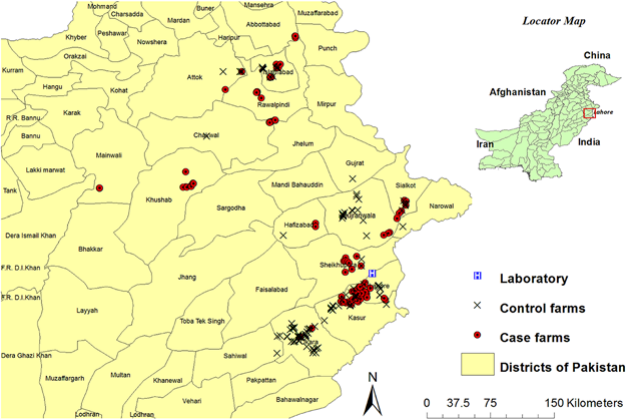
Spatial distribution of case and control farms in different districts of Pakistan, sampled between May 2009 and January 2010.

A total of 133 case farms and matched controls were identified, contacted and visited in the study area. Their positions are marked on the map in Fig. 1 and shows that case farms were mostly concentrated near Lahore district followed by Kasur district. The control farms were mostly situated near Okara and Gujranwala districts though there was overlap.

A total of 34 variables were screened in the univariable analysis and 25 were associated with being a case or control (Table 1). Among these, 14 factors were found to be associated with an increase in the odds of exposure in case farms compared to control farms and included; distance from the main road of ≤0.5 km, distance from the nearest commercial farm of ≤1 km, distance from the nearest case/infected farm of ≤1 km, age of flock at the time of submission of samples to laboratory, presence of pond/canal/water reservoir near the farm wild/migratory birds coming on the pond/canal/water reservoir, history of infection with IBD in the same flock, infection with *Escherichia coli* in the flock, sharing of farm equipment, raising backyard poultry/pet birds on the farm premises, selling of eggs/birds directly to live bird retail stalls, selling of culled birds directly to live bird retail stalls and cover on the water storage tank. Twelve factors were found to be protective i.e. having smaller odds of exposure among farms with AI subtype H9N2 infection as compared to controls.

**Table 1:** Result of univariable analysis by Mantel-Haenszel matched pair analysis of the possible risk factors associated with the risk of AIV H9N2 infection among commercial poultry farms.

Variable	Variable level	Case (n = 133)	Control (n = 133)	M-H Odds Ratio (OR) and 95% CI	p-value*
Distance from the main road	≤0.5 km	128	118	3.50 (1.15–10.6)	0.027
	>0.5 km	5	15	1	
Distance from the nearest commercial farm	≤1 km	85	48	1.80 (1.04–3.12)	0.035
	>1 km	69	64	1	
Distance to the other case/infected farm	≤1 km	99	14	29.3 (9.28–92.7)	<0.001
	>1 km	34	119	1	
Pond/canal/water reservoir near the farm	Yes	128	116	7.00 (1.59–30.8)	0.010
	No	5	17	1	
Wild/migratory birds coming on the pond/canal/water reservoir	No water reservoir	5	17	1	
	Yes	126	114	7.00 (1.59–36.8)	0.01
	No	2	2	7.00 (0.60–81.7)	0.12
Farm fully fenced	Yes	96	115	0.41 (0.21–0.77)	0.006
	No	37	18	1	
Age of flock at the time of submission of samples	≤50 days	44	27	2.13 (1.16–3.94)	0.015
	>50 days	89	106	1	
History of Infectious Bursal Disease in the flock	Yes	96	57	3.05 (1.82–5.13)	<0.001
	No	37	76	1	
Infection with *Escherichia coli* in the flock	Yes	113	100	1.86 (0.95–3.49)	0.051
	No	20	33	1	
Disinfection of areas around sheds	Yes	64	87	0.39 (0.22–0.72)	0.002
	No	69	46	1	
Sharing of farm equipment	Yes	15	6	2.50 (0.97–6.44)	0.057
	No	118	127	1	
Raising backyard/pet birds on farm premises	Yes	25	4	8.00 (2.91–26.4)	<0.001
	No	108	129	1	
Selling of birds/eggs directly to the live bird retail stalls	Yes	49	12	7.16 (3.05–16.8)	<0.001
	No	84	121	1	
Selling of culled birds directly to live bird retail shop	Yes	30	15	2.87 (1.29–6.43)	0.010
	No	103	118	1	
Dipping area/footbath at the farm entrance	Yes	31	49	0.49 (0.27–0.87)	0.014
	No	102	84	1	
Worker change/disinfect boots	Yes	53	78	0.42 (0.24–0.73)	0.002
	No	80	55	1	
Worker changed cloths	Yes	40	71	0.33 (0.18–0.58)	<0.001
	No	93	62	1	
Visitor change/disinfect boots	Yes	56	77	0.50 (0.30–0.84)	0.009
	No	77	56	1	
Cover on the water tank reservoir	No water tank	5	5	1	
	Yes	89	125	0.83 (0.23–3.02)	0.778
	No	39	3	10.8 (1.95–60.5)	0.006
Disposal of dead birds	Properly	38	59	0.43 (0.24–0.78)	0.005
	Not properly	95	74	1	
Type of Shed	Open sheds	73	44	1	
	Window- less	53	74	0.35 (0.19–0.66)	0.011
	Semi-window-less	7	15	0.30 (0.12–0.81)	0.016
Ventilation system	Fan ventilated	60	90	0.33 (0.19–0.60)	<0.001
	Naturally	73	43	1	
Drinking water system	Automated	54	74	0.46 (0.26–0.82)	0.008
	Manual	79	59	1	
Feeding system	Automated	46	69	0.48 (0.28–0.81)	0.006
	Manual	87	64	1	
Floor cover type	Concrete	71	47	0.42 (0.24–0.73)	0.002
	Saw dust	62	86	1	

*P-value based on Wald statistic

The collinearity between these variables was assessed and plotted (S1 Fig. Correlation plot for all variables that passed the initial univariable screening). There was clearly considerable collinearity between a number of variables, all related to biosecurity/disease management. Using PCA and kmeans clustering, 12 of the variables (Farm fully fenced, disinfection of area around sheds, sharing farm equipment, footbath/dipping areas on the farm, worker changed boot, worker changed cloths, visitors changed boot, ventilation system, floor cover type, drinking water system, disposal of dead birds) were collapsed into a new variable named ‘biosecurity’ that was used in the final modeling to replace all these correlated variables.

An initial model built using backward elimination is given in Table 2. Four variables remained in the model, which were, being less than 1 km from a case/infected farm; being less than 1 km from other nearest commercial poultry farm; having a history of IBD on the farm and selling eggs directly to live bird retail stalls. Biologically these are all plausible. This model was compared with one generated by the automated stepwise AIC procedure which produced a similar but more complex model which substituted IBD with type of shed, having cover on water storage tank and biosecurity (details not shown). However, the OR and 95% confidence intervals of the distance to the nearest case/infected farm were both extremely large and wide. This is partly because, not surprisingly, being near an case/infected farm is highly risky and strongly associated such that when the 2x2 table for the matched data was examined, there were only 3 discordant case control pairs in one cell which makes precision of the estimate difficult to achieve.

**Table 2:** Model 1 — Risk factors associated with Avian Influenza type H9N2 infection on commercial poultry farms in Pakistan based on a backward manual stepwise selection process on all variables available in Table 1.

Potential risk factors	Response levels	Regression coefficient	Standard error	OR	95% CI for OR	p-value
Distance from the nearest commercial farm	>1km			1		
	≤1km	-1.3082	0.6068	0.270^a^	0.082–0.888	0.031
Distance from the nearest case/infected farm	>1km			1		
	≤1km	3.7927	0.7690	44.4^b^	9.83–200	<0.001
History of Infectious Bursal Disease in the flock	No			1		
	Yes	1.1753	0.4701	3.20^c^	1.29–8.14	0.012
Selling of birds/eggs directly to the live bird retail stalls	No			1		
	Yes	2.0397	0.6828	7.69^d^	2.02–29.3	0.002

R^2^ = 0.365 (out of a possible 0.5)

^a^The odds of being near the other commercial farm<1km was 0.270 (CI 95%: 0.082–0.888) times less in case farm than the odds of exposure in the control farms

^b^ The odds of being near the case/other infected farm<1km was 44.4 (CI 95%: 9.83–200) times greater than the odds of exposure in the control farms.

^c^The odds of having history of infection with IBD for case was 3.20 (95% CI: 1.29–8.14) times greater than the odds of exposure in the control farm.

^d^The odds of selling the birds/and or eggs directly to live bird retail shops was 7.69 (CI 95%: 2.02–29.3) times more than the odds of exposure in the control farms.

In order to check the impact of this variable, we dropped it and reran a backward model and a new model (Model 2) was developed (Table 3). Interestingly although the variable ‘having cover for water storage tanks’ does not appear significant based on the Wald statistic p-value, dropping it seems to have a large impact on the fit based on the R^2^ decreasing. This was checked by running an automated model selection algorithm based on the AIC which produced the same model. A manual forward selection process produced a slightly simpler model with just the IBD and selling eggs to live bird stalls staying in the final model (not shown).

**Table 3:** Model 2 — Risk factors associated with Avian Influenza type H9N2 infection on commercial poultry farms in Pakistan based on a backward manual stepwise selection process on all variables available in Table 1 except distance to the nearest case farm.

Potential risk factors	Levels	Regression coefficient	Standard error	Odds ratios	95% CI for OR	p-value
Distance from the main road	>0.5km			1		
	≤0.5km	1.5244	0.7328	4.59^a^	1.09–19.3	0.038
Cover on the water tank	No water tank			1		
	No cover	0.8490	0.9830	2.34	0.34–16.1	0.388
	Yes cover	-1.1604	0.7767	0.3130 ^b*¶^	0.068–1.44	0.135
History of Infectious Bursal Disease in the flock	No			1		
	Yes	1.0979	0.3741	3.00^c^	1.44–6.24	0.003
Selling of birds/eggs directly to the live bird retail stalls	No			1		
	Yes	2.3598	0.5847	10.6^d^	3.37–33.3	<0.001

R^2^ = 0.235 (out of possible 0.5)

^a^The odds of having the distance from the main road <0.5km was 4.59 (CI 95%: 1.09–19.3) times higher than the odds of exposure in the control farms.

^b*¶^Non significant protective factor, i.e odds of having cover on the water storage tank on case farm was 0.313 (CI 95%: 0.068–1.44) times less than the odds of having cover on the water storage tank on control farms.

^c^The odds of having history of infection with IBD for case was 3.00 (95% CI: 1.44–6.24) times greater than the odds of exposure in the control farm.

^d^The odds of selling the birds/and or eggs directly to live bird retail shops was 10.6 (CI 95%: 3.37–33.3) times more than the odds of exposure in the control farms.

## Discussion

Model selection is not a precise science as there is no one “true” model and different approaches/algorithms will give different models. Although automated approaches from machine learning theory are increasingly being used in epidemiology, they have the disadvantages that they cannot deal with subtleties of variable quality and tend to over fit to a given dataset making generalization difficult. The researcher is therefore left to look at as many modeling approaches as possible and distill the complexity to identify the core variables. The analysis presented in this study suggests regardless of the modeling approach that selling birds to live bird retail stalls is strongly associated with an increased risk of AIV H2N9 on the farm. Furthermore, previous history of IBD also came through in the models, as did having cover for water storage tanks. Distance to a case/infected farm, other commercial farms or the main road appears to all have some potential association, as does biosecurity.

Selling of eggs or birds directly to live bird retail stalls appeared to be strongly associated (OR = 9.76; model 1) with an increased risk of AI in the current analysis. When farmers sell their products to live bird retail markets, the stall owner either picks the birds, or farm vehicles deliver the products to retail stalls. The movement of individuals and vehicles between farms and markets might spread the virus mechanically.

A farm having a previous history of infection with IBD was more likely to become infected with AI (OR = 3.2; model 1). There is very little information available about the potential effects of previous exposure to IBD virus and the subsequent susceptibility to AIV infection and/or associated clinical signs, lesions, and virus shedding. Ramirez-Nieto et al. [35] have reported the results of their experiment, which clearly indicate that prior exposure to IBD virus may increase the chances of AIV infection in chicken. They demonstrated that pre-existing conditions, such as exposure to IBD virus, contribute to the mechanism of adaptation and generation of AIV strains with altered host range, tissue tropism, or virulence. They recommended that the predisposing factors, such as IBD virus infection, should be considered amongst the risk factors for the emergence of AIVs with increased pathogenic potential.

Having cover on the water storage tank though was not strongly associated with a decrease in the risk of AI (OR = 0.313; p>0.05). The presence of a cover on a water tank is thought to prevent the transmission of AI virus, which might occur when droppings and other excretions of wild birds contaminate the water tank used for housed poultry.

Distance of the poultry farm to a main road of ≤0.5 km may increase the risk of AI subtype H9N2 infection. Many studies have been conducted [15,16, 19], which demonstrated that proximity to major roads was associated with avian influenza.

Short buffer distance between farms elevates poultry density within a given radius and could enhance the risk of spread of avian influenza [21]. The multivariable analysis in the current study suggests that the risk of AI subtype H9N2 infections was not increased with decreasing distance to the nearest commercial farm or increasing poultry density, which is usually speculated. There may be other factors, such as climate factors or biosecurity levels on farms, which influenced the spread of the virus and reduced the likelihood of becoming infected based on only the distance from the infected farms or contaminated places. Distance to the nearest case/infected farm of ≤1 km was strongly associated with an increased risk of AIV. Causally this factor plays an important role in the spread of infection, both as windborne spread, if the distance between farms is less than 50 meters [27] and mechanical transfer by other routes e.g. human movement between farms [13]. The large effect size and the wide CIs were a concern for this variable, not because the association may not be true, but because of the effect on the model and the estimation process where there are cells with small numbers of observations affecting precision.

Ponds, canals or water reservoirs can play an important role in the introduction of AI because these attract wild birds [16, 23], which may then contaminate the environment [17, 28]. Further wild birds may directly contaminate poultry sheds if they have access, acting as mechanical and/or biological vectors (shedding the virus in droppings) and are important particularly for introducing virus to new areas [23, 28]. There were problems with collinarity with presence of pond/canals/water reservoirs near farm and wild birds coming to that pond and so these two variables were tried in separate modeling runs but neither stayed in any of the final models.

A number of variables related to biosecurity measures were collapsed using PCA and kmeans clustering. Good biosecurity measures such as a farm fence which can keep wild and stray dogs, cats, foxes, pigs and other wild animals away from the susceptible birds and prevent mechanical transmission [29–31] and potentially limit contact with wild birds [28, 32]; use of footbath/disinfectant; workers changed/disinfected boots before entering the sheds; worker changed cloths before entering the sheds; visitor changed/disinfected boots before entering the sheds; presence of dipping area/footbath at the entrance of farm and sharing equipment were all associated with AIV even after collapsing into a single variable. The main visitors who entered the farm areas are farm services crews (debeaking staff, vaccination crew, bird catchers, veterinarian, farm consultant and owner of the farms) [13, 21]. Similarly in Pakistan, poultry farmers who apply strict biosecurity measures sometimes relax these rules for visitors, who enter the bird areas. However, biosecurity variable was only retained in the more complex model based on AIC (not shown).

Age of flock (in days) at the time of submission of samples to the laboratory was considered a potential confounder as clearly older flocks have had more time to be exposed and although it showed a positive association with AIV infection in the univariable analysis, was non-significant in the final model. Increase in age, increases the susceptibility of birds (due to housing stresses and the associated negative effects on immune status) and increases the opportunity for virus exposure [34].

*E*. *coli* has been observed to aggravate the clinical condition of birds earlier infected with AIV subtype H9N2 [52], however in the current study a history of infection with *E*. *coli* in the flock was not associated with an increased risk of AI infection.

A number of other management factors reported in other studies were also examined in the current study including the proper disposal of dead birds [10, 21], trucks/vehicles entering the farm premises [36], the presence of automatic fan ventilation system [10], the presence of rodents in the sheds and keeping backyard poultry on the same premises [37, 38]. Although several of these were associated with AI subtype H9N2 infection in the univariable analysis, none were retained in the final model. Epidemiological tracing has shown that vehicles visiting farm premises for different purposes are one of the routes of introduction of virus [36]. Capua et al. [53] have also reported an association between the spread of AI virus and different type of vehicles visiting the farms (e.g., feed trucks and abattoir delivery etc., which frequently visit a number of farms daily, regardless of the species reared and of the type of production).

### Limitations of the Study

There was no complete database of poultry farms in Pakistan and cases and controls were selected from the logbook of a local commercial laboratory. Selection bias due to non-random sampling could occur because most of the farms were physically close to this laboratory and were frequently submitting their samples for diagnostic purposes and had higher probabilities of being selected as cases or control. Also it is possible that the better managed farms were biasly selected because they were more likely to be investigating disease problems and this may impact the study ability to identify management risk factors. The number of farms, which were situated several miles away from the laboratory, was high but very few of them submitted samples for diagnostic purposes due to long distance between farm and laboratory. Due to this reason they were under represented in the data set. This could lead to healthcare access bias as only those farms were submitting samples that have easy access to laboratory.

The control farms were selected on the basis of absence of clinical signs of influenza on the day of selection. However, the likelihood of a previous episode of disease could not be established with certainty with this criterion, this could serve as a possible source of selection bias in the study. Ideally flocks would have been confirmed negative by diagnostic tests. but resources were not available to do this in this setting. Due to these selection biases the exposure effect may be underestimated or overestimated.

In addition, respondents may have interpreted some questions subjectively. For example, when the respondents of the farms were asked the question “Have you seen wild birds inside the shed”, most of them may have interpreted it as visual confirmation of the presence of wild bird inside the shed and because some of them who did not see wild birds inside the shed themselves, may have answered “No” to this question. The same applies to the question relating to the presence of rodents inside the shed. Most of the respondents answered this question on the basis of personal evidence of seeing rodents inside the shed. Similarly when they were asked that if they have seen wild/migratory birds coming to the nearby pond/water body, some respondents were confused by the type or species of bird; for example, some of them considered crow as wild bird and replied “Yes”, to the question while in other instances, some respondent didn’t consider crow as a wild bird and replied “No” to this question. The questions could have been worded more precisely to avoid these confusions. Some respondents also tried to hide or gave misleading information, for example when they were asked, “Do they cover the water storage tanks”. They might have considered that just putting a half cover on the tank is sufficient and replied, “Yes”, while in some cases practically there was no complete cover on the tanks. In some instances the response might have been influenced by recall bias of the respondent.

### Conclusions

The aim of this study was to identify potential risk factors and quantify their association with the presence of low pathogenic AI subtype H9N2 among commercial poultry farms in Pakistan. The current study has identified a set of similar risk factors to those identified in other studies from other countries e.g. USA, China, Japan, Hong Kong, Bangladesh, Vietnam, Thailand and Pakistan, which shows that these well-known factors are mainly responsible for increasing risk of AIV infection. Control of these risk factors could possibly reduce the risk of AIV infection in commercial poultry farms in Pakistan and in other developing countries, particularly in South Asia. Particular attention is needed for the control of risk factors that are related to movement of humans. Good management practices and strict biosecurity rules together can prevent the entry of infection to farms and transmission within sheds on a farm. Some examples of these practices include banning the sale of eggs/birds directly to retail stalls, increasing the distance between two commercial farms (more than 1 km), and covering water sources to prevent wild bird access. Proper vaccination against IBD is also important for the better control of diseases like AIV.

## Supporting Information

S1 Fig Correlation plot for all variables that passed the initial univariable screening. The variables have been reordered in the plot to group highly correlated variables together.
(TIF)

S1 Appendix Questionnaire for the case-control study to identify risk factors associated with avian influenza subtype H9N2 on commercial poultry farms in Pakistan. (PDF)
