# Supplementary figures and images for: A Case-Control Study to Identify Risk Factors Associated with Avian Influenza Subtype H9N2 on Commercial Poultry Farms in Pakistan
Source: PLoS One. 2015 Mar 16;10(3):e0119019. doi: 10.1371/journal.pone.0119019 (PMC4361405; doi:10.1371/journal.pone.0119019)

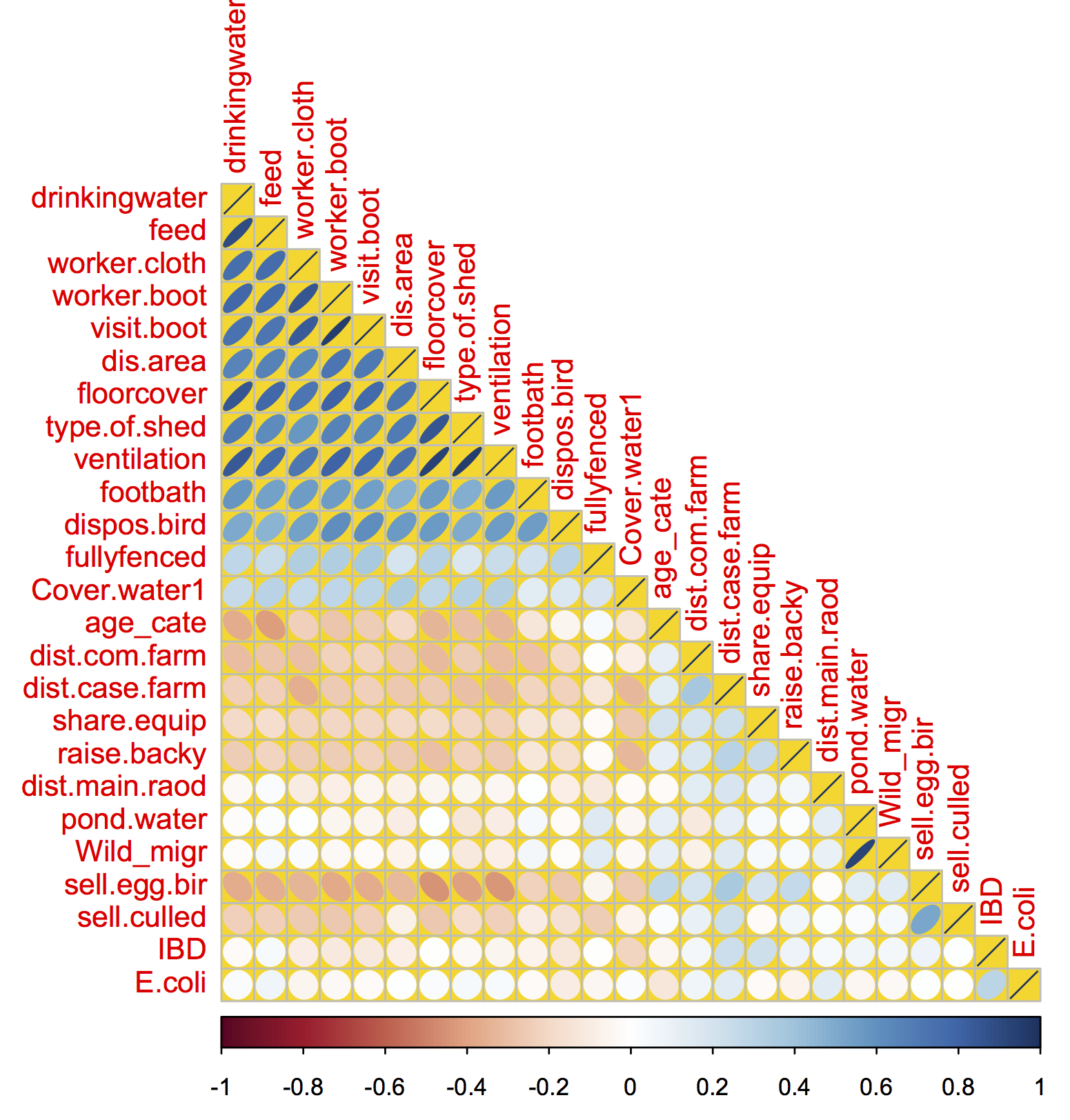

Supplement: S1 Fig — The variables have been reordered in the plot to group highly correlated variables together. (TIF) [file pone.0119019.s001.tif]
