# Supplementary material for: A Case-Control Study to Identify Risk Factors Associated with Avian Influenza Subtype H9N2 on Commercial Poultry Farms in Pakistan
Source: PLoS One. 2015 Mar 16;10(3):e0119019. doi: 10.1371/journal.pone.0119019 (PMC4361405; doi:10.1371/journal.pone.0119019)
Supplement: S1 Appendix — (PDF) [file pone.0119019.s002.pdf]

Technical Appendix S1

Questionnaire for the case-control study to identify risk factors associated with avian influenza subtype H9N2 on commercial poultry farms in Pakistan

- **This section must be filled in before completing the questionnaire:**

Date:	Questionnaire No.:
District:	Farm Code:
Land Area / Farm Area (Sq. meter) :	

- **Tick only one answer:**

SECTION 1 (LOCATION OF FARM)

Latitude:	Longitude:

1. Distance of the farm from main road:.......................... ________________ (km)
2. Distance of farm from nearest commercial poultry farm:............. ________ (km)
3. Distance from nearest case (infected) farm: ....________________ (km)
4. Is this a rice/wheat growing area? .... **Yes □ No □**

SECTION 2 (BIOSECURITY)

---

5. Is there a pond (s)/water reservoir inside/near your farm? .... **Yes □ No □**
6. If yes, do you see any wild/migratory birds come to the pond(s)/reservoir? ………………………………………………………………........ **Yes □ No □**
7. Can wild birds enter into the shed? .... **Yes □ No □**
8. Is the farm fully fenced to prevent entry of wild animals? ……...… **Yes □ No □**
9. Do you disinfect the area around sheds? .... **Yes □ No □**

10. If yes, how often do you disinfect the area around sheds?
.... **regularly □ not regularly □**

11. Do you wash and disinfect the cages before re-introduction into the farm?
……………………………………………………………........………... **Yes □ No □**

12. Do you share the farm equipment with other farms? .... **Yes □ No □**

13. Do the workers change rubber boots or soak them in foot bath before entering the bird area? ....…… **Yes □ No □**

14. Do the workers change cloths or spray disinfectant on them before entering the bird area? .... …..… **Yes □ No □**

15. Do visitors change rubber boots or soak them in foot bath before entering the bird area? .... **Yes □ No □**

16. Do you have foot bath/dipping area at the entrance of farm? …….. **Yes □ No □**

SECTION 3 (FLOCK HISTORY)

---

17. What was the mortality (%) on your farms? .... ____________________

18. Do you vaccinate your birds against H9N2 AIV strain? .... **Yes □ No □**

19. Age of flock: .... _______________ (days)

20. Capacity of farm: .... _________ (no. of birds)

21. Was the flock affected by infectious bursal disease? .... **Yes □ No □**

22. Was the flock affected by E. Coli infection? .... **Yes □ No □**

SECTION 4 (FARM MANAGEMENT)

---

23. Do you raise backyard poultry/pet birds at your farm? .... **Yes □ No □**

24. Do you see rodents in the sheds? .... **Yes □ No □**

25. Can truck/large vehicle enter the farm? .... **Yes □ No □**

26. Do you sell your birds/eggs directly to retail shop? .... **Yes □ No □**

27. Do you sell culled birds directly to retail shop? .... **Yes □ No □**

28. Do you store water? .... **Yes □ No □**

29. If yes, do you have cover for your water tank(s)? .... **Yes □ No □**

30. How do you dispose waste from shed after cleaning?..

......................................……**properly disposed of □ not properly disposed of □**

31. How do you dispose of the dead birds?..

……………………....………**properly disposed of □ not properly disposed of □**

32. Which type of sheds do you have…..**windowless □ open □ semi- windowless □**

33. What kind of ventilation system is used on your farm? ....

.... **fan ventilation □ natural □**

34. How does your flock get their drinking water? ……........ **automatic □ manual □**

35. How does your flock get their feed? .... **automatic □ manual □**

36. What kind of floor cover is in the sheds? .... **concrete □ saw dust □**

37. Egg/bird catching collection system: .... **automatic □ manual □**
